# Outcomes of Underwater Endoscopic Mucosal Resection for Giant Colorectal Polyps: A Multicenter Study

**DOI:** 10.1055/a-2910-5182

**Published:** 2026-08-03

**Authors:** Rahul Karna, Natalie Wilson, Amir S. Seid, Kaylee Larsen, Thomas Enke, Ali Hassam, Anand R. Kumar, Shawn L. Shah, Joy Zhao, Alyssa Y. Choi, Danny Issa, Ahmed Saeed, Jennifer Phan, Jeffrey D. Mosko, Frances Dang, Anders Westanmo, Andrew Reinink, Jason B. Samarasena, Aasma Shaukat, Mohammad Bilal

**Affiliations:** 1 Division of Gastroenterology and Hepatology 5635 University of MinnesotaMinneapolisMinnesotaUnited States; 2 Department of Pharmacy 20040 Minneapolis VA Medical CenterMinneapolisMinnesotaUnited States; 3 Division of Gastroenterology and Hepatology 6797 The University of North Carolina at Chapel Hill School of MedicineChapel HillNorth CarolinaUnited States; 4 Division of Gastroenterology and Hepatology 6915 Mayo ClinicRochesterMinnesotaUnited States; 5 Department of Internal Medicine 129263 University of Colorado Anschutz Medical CampusAuroraColoradoUnited States; 6 Division of Gastroenterology and Hepatology 129263 University of Colorado Anschutz Medical CampusAuroraColoradoUnited States; 7 Division of Gastroenterology and Hepatology 3627 East Carolina UniversityGreenvilleNorth CarolinaUnited States; 8 Division of Gastroenterology and Hepatology 6529 Thomas Jefferson University HospitalsPhiladelphiaPennsylvaniaUnited States; 9 Division of Gastroenterology and Hepatology 20115 Dallas VA Medical CenterDallasTexasUnited States; 10 Department of Internal Medicine 6559 Thomas Jefferson UniversityPhiladelphiaPennsylvaniaUnited States; 11 Division of Gastroenterology and Hepatology 20128 VA Puget Sound Health CareSeattleWashingtonUnited States; 12 Division of Gastroenterology and Hepatology 12222 University of California Los Angeles David Geffen School of MedicineLos AngelesCaliforniaUnited States; 13 Division of Gastroenterology and Hepatology 486147 AdventHealth Centra Care KansasShawneeKansasUnited States; 14 Division of Gastroenterology and Hepatology 709196 Hoag Digestive Health InstituteNewport BeachCaliforniaUnited States; 15 Division of Gastroenterology and Hepatology 10071 St Michael’s HospitalTorontoCanada; 16 Division of Gastroenterology and Hepatology 8788 University of California IrvineIrvineCaliforniaUnited States; 17 Division of Gastroenterology & Hepatology 20040 Minneapolis VA Medical CenterMinneapolisMinnesotaUnited States; 18 Division of Gastroenterology & Hepatology 8788 University of California IrvineIrvineCaliforniaUnited States; 19 12296 NYU Grossman School of MedicineNew YorkNew YorkUnited States; 20 Division of Gastroenterology and Hepatology 129263 University of Colorado Anschutz Medical CampusAuroraColoradoUnited States

**Keywords:** underwater endoscopic mucosal resection, large non-pedunculated colorectal polyps, recurrence, outcomes

## Abstract

**Introduction** Underwater endoscopic mucosal resection (uEMR) technique has gained popularity for removal of large non-pedunculated colorectal polyps (LNPCPs) due to high en-bloc resection rates and lower recurrence rates. However, there is no information regarding outcomes of uEMR for giant colorectal polyps. Therefore, we aimed to evaluate the outcomes of uEMR for giant colorectal polyps across multiple centers in North America.

**Methods** We conducted a multicenter retrospective study of adults with giant colorectal polyps undergoing uEMR between January 2019 and November 2025. Giant colorectal polyps were defined as LNPCPs ≥40 mm. Primary outcomes were technical success and rate of recurrent adenoma. Secondary outcomes were severe adverse events (SAEs) and predictors of technical success, recurrence, and SAEs.

**Results** A total of 137 polyps, with mean polyp size 47.6 mm were included. Majority of the lesions were located in right colon (61%, *n* = 83) with the most common histopathology being high-grade dysplasia (HGD) (31.4%, *n* = 43). Technical success was achieved in 90.5% ( *n* = 124) with en-bloc resection rate of 11.7% ( *n* = 16). Polyp fibrosis (OR: 0.08) and HGD (OR: 0.15) were independent predictors of inability to achieve technical success. Of 92 (67.1%) patients with follow-up, recurrent polyp was present in 8.7% ( *n* = 8). SAEs were noted in 9.5%, consisting of delayed bleeding (9.5%, *n* = 13), while no delayed perforation or post polypectomy syndrome was noted.

**Discussion** Our study demonstrates that uEMR is technically feasible for the removal of giant colorectal polyps with an acceptable safety profile and recurrence rate.

## Introduction

Colorectal cancer (CRC) is the third-most common cancer and second leading cause of cancer-related deaths in the United States. 1 Endoscopic detection and resection of pre-malignant polyps can lead to a reduction in CRC incidence and mortality. 2 3 The United States Multi-Society Task force (USMSTF) on colorectal cancer recommends endoscopic mucosal resection (EMR) for removal of large non-pedunculated colorectal polyps ≥20 mm in size (LNPCPs). 4

Typically, EMR is performed using a submucosal injectate to lift the polyp and separate the polyp from the muscularis propria and then using a snare with electrocautery to resect the polyp in a single piece (en-bloc resection) or in multiple pieces (piecemeal resection). 5 A systematic review and meta-analysis of 50 studies with 6779 colorectal polyps ≥20 mm demonstrated an excellent safety profile of endoscopic resection with a serious adverse event (SAEs) rate of 1%, but noncurative resection and recurrence rates of 8–14%, respectively. 6

Underwater endoscopic mucosal resection (uEMR) is rapidly emerging as a popular alternate technique for removing LNPCPs. This technique utilizes the buoyancy effect of saline immersion to allow mucosa and submucosa to float away from the muscularis propria layer. First described by Binmoeller et al. in 2012, this technique obviates the need for submucosal lift. 7 Multiple studies and meta-analysis have evaluated the safety and efficacy of uEMR compared to conventional or hot-EMR (h-EMR). 8 9 10 11 12 A recent prospective randomized trial evaluated h-EMR and uEMR for removal of colorectal polyps 20–40 mm in size and found a significantly higher en-bloc resection rate (33.3% vs. 18.4%), R0 resection rate (32.1% vs. 15.8%), and lower resection time (8 min. vs. 14 min.), with a similar safety profile of uEMR compared to h-EMR. 13 The recurrence rate with uEMR was 15.1% as compared to 24.6% with h-EMR ( *p* = 0.25). In a subgroup analysis of polyps between >30 and 40 mm in size, the recurrence rate was found to be significantly lower for in the uEMR group (6.3% vs 42.9%). 13 Previously, meta-analysis demonstrated improved en-bloc resection, lower rates of incomplete resection, and recurrence rate with uEMR compared with h-EMR for LNPCPs > 20 mm in size. 8 9

Despite the increasing popularity of uEMR, the current data for using uEMR for removal of LNPCPs is limited to polyps <40 mm in size. Therefore, the aim of the present study was to evaluate the safety, efficacy, and outcomes of uEMR for giant colorectal polyps (LNPCPs ≥40 mm) across multiple centers in North America.

## Materials and Methods

### Study Design and Population

**Fig. 1:**
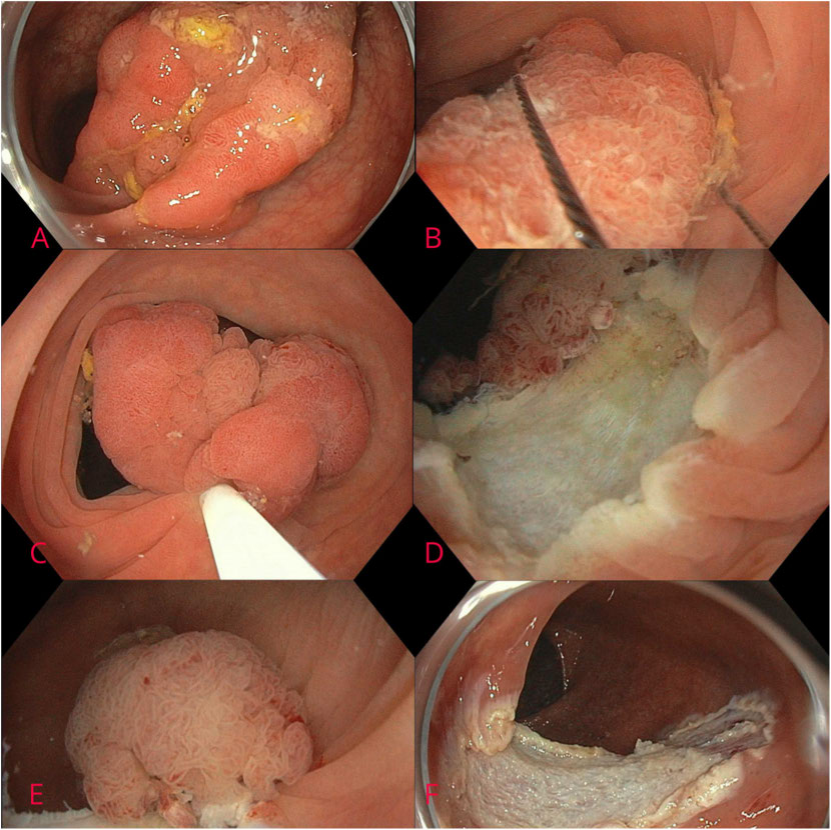
Piecemeal underwater endoscopic mucosal resection (uEMR) technique. ( **A** ) Visual inspection of the polyp is performed. ( **B** ) The lumen is then instilled with saline and the snare is opened around the polyp. ( **C** ) Once the polyp is within the snare, the snare is slowly closed around the polyp, and the polyp is then tented away from the underlying muscularis propria. ( **D** ) Electrocautery is applied to resect the polyp, and the resection bed is inspected to assess for perforation and residual polyp. ( **E** ) The process is repeated until the entire polyp has been resected. ( **F** ) The final post resection bed.

We conducted a multicenter retrospective study of patients who underwent uEMR for colorectal polyps across 10 centers in North America between January 2019 and November 2025. All adult patients who underwent uEMR of giant colorectal polyps were included. Giant colorectal polyps were defined as LNPCPs ≥40 mm. 14 Lesions were carefully examined under white light endoscopy followed by narrow band imaging to assess features suggestive of submucosally invasive CRC. All endoscopists involved in the study had >5 years’ experience of performing complex endoscopic resection. Large lesions with morphological features predictive of submucosal invasion were referred for ESD or surgery as appropriate and excluded from the study. Fig. 1 demonstrates steps involved in removal of LNPCPs using uEMR. Patients with inflammatory bowel disease, those with polyp <40 mm in size, or polyps where resection was performed with techniques other than uEMR were also excluded. This study was approved by the institutional review board of Minneapolis Veterans Affairs Medical Center (primary institution). Institutional review board approval was also obtained at participating sites or a data use agreement was obtained between primary institution and each participating center.

Data on characteristics of patients including age, antiplatelet and anticoagulant use, American Society of Anesthesiologists class, and history of hereditary polyposis syndrome were recorded. Information on polyp characteristics including polyp size, location, morphology (Paris classification), as well as history of prior polyp resection was also recorded. Lesion size was determined by the endoscopist through visual estimate or using snare size or other equipment as a guide per endoscopists’ discretion.

Procedural data such as snare size, margin ablation, defect closure, duration, and cross-over to another technique were recorded. The criteria for lesion selection and decision to perform margin ablation or defect closure or the intraprocedural threshold to cross over to another technique were up to the discretion of the endoscopist performing the procedure. When defect closure was performed, type of closure, such as through-the-scope (TTS) clips; tissue approximation devices like Mantis (Boston Scientific, Marlborough, Mass, USA) or Dual Action Tissue (DAT) clip (Micro-Tech Endoscopy, USA Inc, Ann Arbor, Michigan, USA); use of suturing devices including over the scope suturing system (OTSS, Overstitch; Boston Scientific, Marlborough, Mass, USA), through the scope suturing system (TTSS, X-Tack Endoscopic Helix Tacking System, Boston Scientific, Marlborough, Mass, USA), or a combination of any of these techniques were recorded. Outcome data including presence of submucosal fibrosis, technical success, histopathology, and AEs were also recorded. Submucosal fibrosis was assessed intraprocedurally and postprocedurally after endoscopic resection. The polypectomy defect site was carefully assessed qualitatively for the presence or absence of fibrosis. We did not use the grading of fibrosis (F0/1/2), as these tools have been utilized to assess submucosal dissection during ESD and have not been validated for assessment of fibrosis in EMR. 15 16 Surveillance data were also collected, including time to first follow-up, presence of recurrent adenoma, and number of procedures required for lesion eradication. Patients were followed up in accordance with the USMSTF guidelines, with scheduled follow-up endoscopy at six months for patients undergoing piecemeal resection, three years for en-bloc resection with negative margins (R0 resection), and those with invasive adenocarcinoma and positive margins on histopathology were referred to surgery. 17

### Study Outcomes

Primary outcomes were technical success and rate of recurrent adenoma. Technical success was defined as complete resection of all visible adenomatous tissue with uEMR without use of a cross-over technique. Secondary outcomes were SAEs, rate of en-bloc resection, and predictors of technical success and severe SAEs. Serious AEs were defined as AEs leading to hospitalization, transfusion, repeat endoscopy, embolization, or surgery. The AEs included immediate AEs (bleeding, perforation) and delayed AEs (bleeding, perforation, and postpolypectomy syndrome). Immediate bleeding was defined as intraprocedural bleeding requiring intervention for hemostasis. Immediate perforation was defined as perforation recognized during the procedure. Delayed events (bleeding and perforation) were defined as AEs necessitating hospitalization, transfusion, repeat endoscopy, embolization, or surgery that occurred within 30 days of the procedure. All AEs were graded as per the American Society for Gastrointestinal Endoscopy (ASGE) adverse events in GI endoscopy (AGREE) classification. 18

### Statistical Analyses

All statistical analyses were performed using SPSS version 26.0 (IBM Corp., Armonk, NY). Categorical variables were compared in percentages, and continuous variables as mean ± standard deviation. Comparison between categorical variables was evaluated using the chi-square test or Fisher’s exact test when appropriate. Continuous variables were assessed using an independent *t* test. Univariate analysis was performed to assess the association of variables to outcomes, including technical success, AEs, and recurrence. Given the limited number of outcome events for certain analyses, model complexity was restricted to minimize overfitting. For multivariate analyses with small event counts (e.g., recurrence and delayed bleeding), only variables that were statistically significant on univariate analysis or demonstrated clinical relevance with a trend toward significance were included. In analyses of recurrence and delayed bleeding, multivariable models were limited to a small number of covariates in accordance with accepted events-per-variable principles. Analyses were performed using complete-case methodology; observations with missing data for outcome or model covariates were excluded from the corresponding analyses. Subgroup analyses were performed where indicated. The *p* -value ≤ 0.05 and odds ratios with 95% confidence intervals not crossing 1 were considered statistically significant.

## Results

### Patients and Polyp Characteristics

A total of 137 polyps in 132 patients undergoing uEMR for giant colorectal polyps were included in the study. The median age was 69 years; 75.7% ( *n* = 100) were male. uEMR was performed for 24 polyps (17.5%) in patients receiving anticoagulants, whereas 5 (3.6%) polyps were resected in patients receiving antiplatelet agents. The majority of uEMRs were performed in patients classified as American Society of Anesthesiologists class III (57.4%, *n* = 74). The most common procedure indication was referral for polyp resection (74.4%, *n* = 102) followed by surveillance colonoscopy for a personal history of polyps (10%, *n* = 14). The mean polyp size was 47.6 mm (SD, 10.6 mm), and the most common polyp location was cecum (28.4%, *n* = 39), with right-sided location comprising 61% ( *n* = 83) of the polyps. The most commonly encountered morphology was Paris class Is (39.1%, *n* = 50) and presence of high-grade dysplasia HGD (31.4%, *n* = 43) was the most common histopathological finding. Prior attempts at resection were noted in 11.7% ( *n* = 16) of polyps. Additional patient and polyp characteristics are summarized in Table 1 .

**Table 1:** Baseline characteristics of included patients, procedural and histopathological outcomes.

Characteristic	Value
Age, years, median (IQR)	69 (60–75)
Male sex, *n* (%)	100 (75.7%)
ASA Class, *n* (%) ^#^	
Class I	2 (1.5)
Class II	44 (34.1)
Class III	74 (57.4)
Class IV	9 (6.9)
Anticoagulation use, *n* (%) ^##^	24 (17.5)
Antiplatelet use (excluding aspirin), *n* (%) ^##^	5 (3.6)
Indication of procedure	
Iron deficiency anemia	3 (2.2)
Colon polyp	102 (74.4)
History of colon cancer	1 (0.7)
Positive FIT/Cologuard	7 (5.1)
Screening colonoscopy	4 (2.9)
History of polyp	14 (10.2)
Other	6 (4.3)
Polyp size, mm, mean (SD)	47.6 (10.6)
Prior resection attempts, *n* (%)	16 (11.7)
Right colon location, *n* (%)	83 (60.6)
Polyp location, *n* (%)	
Cecum	39 (28.4)
Ascending	33 (24.1)
Hepatic flexure	11 (8.0)
Transverse	23 (16.8)
Splenic flexure	2 (1.5)
Descending	8 (5.8)
Sigmoid	8 (5.8)
Rectum	13 (9.5)
Polyp morphology (Paris classification)*	
Is	50 (39.1)
Isp	7 (5.5)
IIa	25 (19.5)
IIb	4 (3.1)
IIa+Is	30 (23.4)
IIa+IIb	6 (4.7)
Others†	6 (4.7)
Presence of submucosal fibrosis or scarring, *n* (%)	29 (21.2)
Polyp histology, *n* (%)	
Tubular adenoma	40 (29.2)
Tubulo-villous adenoma	28 (20.4)
Sessile serrated lesion	8 (5.8)
Traditional serrated adenoma	3 (2.2)
Sessile serrated lesions with dysplasia	1 (0.7)
Presence of high-grade dysplasia	43 (31.4)
Invasive adenocarcinoma	12 (8.8)
Others ^†^	2 (1.5)
Procedure duration, median (IQR), minutes	73 (52–102)

*# ASA classification data not available in 3 patients.*

*## Data presented per polyp.*

* Data not available for 9 polyps.

*
† **Others include:** IIa+IIc, IIb+IIc, IIc, IIc+Is, and Is+Isp.
*

### Procedural Details

The median procedure duration was 73 minutes (IQR, 52-102 minutes). Snare diameters used for resection were: 10 mm ( *n* = 10), 15 mm ( *n* = 41), 20 mm ( *n* = 20), ≥ 25 mm ( *n* = 13). The majority of uEMR were performed in a piecemeal fashion (88.3%, *n* = 121). Defect closure was performed in **65.6%** ( *n* = **90)** with majority using through-the-scope clips (56.6%, *n* = 54). Majority of the patients ( **92.7%, *n* = 127** ) underwent margin ablation—either argon plasma coagulation ( *n* = 96) or snare tip soft coagulation ( *n* = 31). Additional procedural characteristics are summarized in Table 2 .

**Table 2:** Details of procedure performed.

Procedural details	*N* = 137
Margin ablation performed	**127 (92.7)**
Snare tip soft coagulation (STSC)	**31 (24.4)**
Argon plasma coagulation (APC)	**96 (75.6)**
Defect closure	**90 (65.6)**
TTS clips	**54 (56.6)**
OTSC	**2 (2.2)**
X-tack	**6 (6.6)**
X-tack + TTS clips	**13 (14.4)**
Approximation device + TTS clip	**14 (15.5)**
Other	**2 (2.2)**
Defect closure stratified by location	
Right colon	**57 (68.6)**
Left colon	**35 (61.1)**
Procedure duration (min), mean (SD)	**78.1 ± 40.6**
Technical success	**124 (90.5)**
En bloc resection	**16 (11.7)**
Crossover to other resection technique*	**10 (7.3)**
APC	**1 (11.1)**
EFTR	**3 (33.3)**
Hot EMR	**4 (44.4)**
Hot avulsion	**1 (11.1)**
Follow up completed	**92 (67.1)**
Time to first follow up (days), median (IQR)	**182 (104–221)**
Time to most recent follow up (days), median (IQR)	**199 (166–295)**
Recurrent polyp	**8 of 92 (8.7)**
Management of recurrent polyps**	**N = 8**
Surgery referral	**2 (25.0)**
Endoscopic resection	**5 (62.5)**
Hot snare	**1 (20.0)**
Cold avulsion + STSC	**2 (40.0)**
uEMR	**1 (20.0)**
Full-thickness resection device	**1 (20.0)**
Endoscopic eradication after 2nd procedure ( *n* = 1)#	**1 (100)**
Awaiting follow up after 2nd procedure	**3 (60)**

*Crossover technique type data unavailable for one patient ; subtype percentages calculated among 9 patients with available data.

**One patient lost to follow-up/data not available.

***# Of five patients undergoing repeat endoscopic resection, one was lost to follow-up and three are awaiting second follow-up.***

### Study Outcomes

#### Technical Success and Predictors of Technical Success

Technical success was achieved in 90.5% ( *n* = 124) with an en-bloc resection rate of 11.7% ( *n* = 16). Mean polyp size of lesions undergoing en-bloc resection was 44.1 mm (SD, 6.9 mm).

Ten patients underwent cross-over technique with h-EMR (requiring submucosal injection with a lifting solution) ( *n* = 4), endoscopic full thickness resection ( *n* = 3), hot avulsion ( *n* = 1) and APC ( *n* = 1) and data not available for one patient. On univariate analysis, technical success of uEMR was lower in polyps with underlying fibrosis compared to those without fibrosis (69.0% vs 96.3%, *p* < 0.001), those requiring cross-over to other techniques compared to those not requiring cross-over techniques (0% vs 98.4%, *p* < 0.001), and those polyps with HGD compared to those without HGD (82.5% vs. 96.2%, *p* = 0.02). Multivariate logistic regression revealed submucosal fibrosis (OR: 0.08, 95% CI: 0.02–0.34, *p* < 0.001) and HGD (OR: 0.15, 95% CI: 0.03–0.70, *p* = 0.02) as independent predictors of inability to achieve technical success ( Table 3 ).

**Table 3:** Univariate and multivariate analyses for predictors of technical success.

Variable	Technical success, *n* (%)		*p* -Value
**Univariate analysis**			
Sex, M vs F ( *n* = 100, 37)	92 (92.0)	32 (86.5)	0.337
History of polyposis/hereditary syndrome, yes vs no ( *n* = 3, 134)	2 (66.7)	122 (91.0)	0.260
Anticoagulation, yes vs no ( *n* = 25, 112)	23 (92.0)	101 (90.2)	1.000
Antiplatelet (excluding ASA), yes vs no ( *n* = 5, 132)	5 (100.0)	119 (90.2)	1.000
Prior resection attempts, yes vs no ( *n* = 16, 119)	13 (81.3)	109 (91.6)	0.185
Polyp location, right colon vs rest ( *n* = 83, 54)	75 (90.4)	49 (90.7)	1.000
Fibrosis/scarring, yes vs no ( *n* = 29, 107)	20 (69.0)	103 (96.3)	<0.001
En-bloc resection, yes vs no ( *n* = 16, 121)	15 (93.8)	109 (90.1)	1.000
Crossover to other technique, yes vs no ( *n* = 10, 126)	0 (0.0)	124 (98.4)	<0.001
High-grade dysplasia, yes vs no ( *n* = 57, 78)	47 (82.5)	75 (96.2)	0.015
Defect closure, yes vs no ( *n* = 90, 47)	83 (92.2)	41 (87.2)	0.368
Approximation device, yes vs no ( *n* = 14, 123)	12 (85.7)	112 (91.1)	0.623
Polyp size (mm), mean ± SD, success vs no ( *n* = 124, 13)	47.2 ± 9.9	50.2 ± 13.9	0.323
**Multivariate analysis**			
Fibrosis/scarring	0.08 (0.02–0.34)		0.001
Polyp size (mm)	0.99 (0.93–1.05)		0.717
High-grade dysplasia	0.15 (0.03–0.70)		0.017
Prior resection attempts	0.47 (0.07–2.94)		0.417

*Multivariable logistic regression restricted to n = 132 with complete covariate data.

#### Recurrence

Follow-up was completed in 67.1% patients ( *n* = 92). Polyp recurrence was noted in 8.7% ( *n* = 8) at a median follow-up of 182 days since procedure. Majority of the recurrent polyps were managed successfully with endoscopic resection (62.5%, *n* = 5), while 25% ( *n* = 2) were referred to surgery. Among patients with previously manipulated polyps ( *n* = 16), follow-up was completed in 68.7% ( *n* = 11) and recurrence was noted in 27.3% (3/11). Table 2 demonstrates details of endoscopic management of recurrent polyps. On univariate analysis, lack of technical success with uEMR (30.0% vs 6.1%, *p* = 0.039) was associated with recurrence compared to achieving technical success with uEMR. However, the relationship was not demonstrated on multivariate analysis ( Table 4 ). Margin ablation was not associated with recurrence on univariate analysis (8.1% vs 16.7%, *p* = 0.43).

**Table 4:** Univariate and multivariate analyses for predictors of recurrence.

Variable	Recurrence, *n* (%)		*p* -Value
**Univariate analysis**			
Sex, M vs F ( *n* = 73, 19)	7 (9.6)	1 (5.3)	1.000
History of polyposis/hereditary syndrome, yes vs no ( *n* = 2, 90)	0 (0.0)	8 (8.9)	1.000
Anticoagulation, yes vs no ( *n* = 22, 70)	1 (4.5)	7 (10.0)	0.675
Antiplatelet (excluding ASA), yes vs no ( *n* = 5, 87)	1 (20.0)	7 (8.0)	0.372
ASA class, IV vs I–III ( *n* = 5, 85)	0 (0.0)	7 (8.2)	1.000
Prior resection attempts, yes vs no ( *n* = 11, 80)	3 (27.3)	5 (6.3)	0.053
Polyp location, right colon vs rest ( *n* = 53, 39)	6 (11.3)	2 (5.1)	0.459
Fibrosis/scarring, yes vs no ( *n* = 20, 71)	4 (20.0)	4 (5.6)	0.067
Technical success, yes vs no ( *n* = 82, 10)	5 (6.1)	3 (30.0)	0.039
Crossover to other technique, yes vs no ( *n* = 9, 82)	2 (22.2)	5 (6.1)	0.141
En-bloc resection, yes vs no ( *n* = 6, 86)	0 (0.0)	8 (9.3)	1.000
Defect closure, yes vs no ( *n* = 56, 36)	5 (8.9)	3 (8.3)	1.000
Approximation device, yes vs no ( *n* = 12, 80)	2 (16.7)	6 (7.5)	0.279
Margin ablation, yes vs no ( *n* = 86, 6)	7 (8.1)	1 (16.7)	0.430
High-grade dysplasia, yes vs no ( *n* = 41, 51)	4 (9.8)	4 (7.8)	1.000
Polyp size (mm), mean ± SD, recurrence vs no ( *n* = 8, 84)	50.6 ± 8.6	48.5 ± 11.0	0.595
**Multivariate analysis**			
Prior resection attempt (Yes vs No)	4.25 (0.77–23.31)		0.096
Technical success (Yes vs No)	0.20 (0.04–1.11)		0.065

*Cohort restricted to lesions with completed follow-up (n = 92). Given only 8 recurrence events, multivariable modeling was limited to two clinically relevant predictors to reduce overfitting risk. The results should be interpreted with caution given that the multivariate analysis was performed on outcome with low number of events.

#### Adverse Events (AEs) and Predictors of Serious Adverse Events (SAEs)

A total of 18 (13.1%) intraprocedural AEs were recorded with majority being intraprocedural bleeding (11.7%, *n* = 16) followed by intraprocedural perforation (1.5%, *n* = 2). All intraprocedural bleeding were managed endoscopically with STSC ( *n* = 8), combination ( *n* = 4), coagulation grasper forceps ( *n* = 2), hot biopsy forceps ( *n* = 1), and bipolar cautery ( *n* = 1). All intraprocedural perforations ( *n* = 2) were managed with defect closure with through-the-scope clips.

Delayed SAEs were observed in 13 patients (9.5%) with all being delayed bleeding. Majority of delayed bleeding occurred in cecum ( *n* = 7), followed by ascending colon ( *n* = 3), transverse colon ( *n* = 2), and rectum ( *n* = 1). In all, 53.8% ( *n* = 7) patients underwent repeat colonoscopy for management of delayed bleeding. About 4 patients underwent therapeutic intervention during colonoscopy (hemoclip plus topical hemostatic agent; hemoclips, soft coagulation, epinephrine plus hemoclip). In total, 1 patient with application of epinephrine plus hemoclip failed endoscopic therapy and underwent angioembolization. Majority of the delayed bleeding were classified as AGREE grade IIIa ( *n* = 6), followed by AGREE grade II in 6 and AGREE grade IV in 1 patient. No cases of delayed perforation or postpolypectomy syndrome occurred. Table 5 summarizes AEs associated with the procedure.

**Table 5:** Adverse events and their management during underwater endoscopic mucosal resection of large non-pedunculated colorectal polyps ≥40 mm in size.

Type of adverse events	Endoscopy	Surgery	Conservative
Intra-procedural bleeding, *n* = 16	16	0	0
Intra-procedural perforation, *n* = 2	2	0	0
Delayed bleeding, *n* = 13	7*	0	6
Delayed perforation, *n* = 0	N/A	N/A	N/A

*Four patients underwent therapeutic endoscopic intervention.

Among lesions with delayed bleeding, prophylactic defect closure was performed in 46.1% ( *n* = 6), utilizing TTS clip in the majority ( *n* = 4), followed by over-the-scope clips and others ( *n* = 1 each). There was no difference between the rates of delayed bleeding in lesions with or without prophylactic defect closure (8.2% vs 15.6%, *p* = 0.24), and in patients with margin ablation (10.4% vs 33.3%, *p* = 0.30). Univariate analysis demonstrated delayed bleeding occurred at a higher rate in patients with ASA class IV compared to ASA class I–III (66.7% vs 6.6%, *p* < 0.001), anticoagulants use compared to those not on anticoagulants (24.0% vs 7.5%, *p* = 0.03), and underlying fibrosis compared to those without fibrosis (30.4% vs 6.4%, *p* = 0.004) ( Table 6 ).

**Table 6:** Univariate and multivariate analyses for predictors of serious adverse events (delayed bleeding).

Variable	Delayed bleeding, *n* (%)		*p* -Value
**Univariate analysis**			
Sex, M vs F ( *n* = 91, 27)	8 (8.8)	5 (18.5)	0.171
History of polyposis/hereditary syndrome, yes vs no ( *n* = 3, 115)	0 (0.0)	13 (11.3)	1.000
Anticoagulation, yes vs no ( *n* = 25, 93)	6 (24.0)	7 (7.5)	0.030
Antiplatelet (excluding ASA), yes vs no ( *n* = 5, 113)	1 (20.0)	12 (10.6)	0.448
ASA class, IV vs I–III ( *n* = 9, 106)	6 (66.7)	7 (6.6)	<0.001
Polyp location, right colon vs rest ( *n* = 72, 46)	10 (13.9)	3 (6.5)	0.246
Prior resection attempts, yes vs no ( *n* = 14, 103)	1 (7.1)	12 (11.7)	1.000
Fibrosis/scarring, yes vs no ( *n* = 23, 94)	7 (30.4)	6 (6.4)	0.004
Technical success, yes vs no ( *n* = 106, 12)	10 (9.4)	3 (25.0)	0.128
En-bloc resection, yes vs no ( *n* = 14, 104)	3 (21.4)	10 (9.6)	0.185
High-grade dysplasia, yes vs no ( *n* = 52, 66)	9 (17.3)	4 (6.1)	0.075
Defect closure, yes vs no ( *n* = 73, 45)	6 (8.2)	7 (15.6)	0.238
Approximation device, yes vs no ( *n* = 13, 105)	0 (0.0)	13 (12.4)	0.356
Margin ablation, yes vs no ( *n* = 115, 3)	12 (10.4)	1 (33.3)	0.298
Polyp size (mm), mean ± SD, bleeding vs no ( *n* = 13, 105)	45.4 ± 8.8	48.1 ± 10.6	0.377
**Multivariate analysis**			
ASA class (IV vs I–III)	19.78 (3.29–118.82)		0.001
Anticoagulation use (Yes vs No)	2.64 (0.55–12.68)		0.225
Fibrosis/scarring (Yes vs No)	6.27 (1.44–27.33)		0.014

*Cohort restricted to lesions with known bleeding status (n = 118; 13 delayed bleeds). Multivariable model includes n = 114 owing to listwise deletion of observations with missing ASA or fibrosis status. The results should be interpreted with caution given the multivariate analysis was performed on outcome with low number of events.

## Discussion

Our large multicenter study evaluating outcomes of uEMR in 140 giant colorectal polyps demonstrates a high rate of technical success, delayed SAE rate in 9.5%, and recurrence rate of 8.7%.

Conventional or hot-EMR (h-EMR) has typically been considered the preferred technique for removing LNPCPs without optical features suggestive of submucosal invasive cancer. Our study demonstrates high technical success in removing giant colorectal polyps using uEMR with an en-bloc resection of 12%. Management of giant colorectal polyps remains challenging due to the risk of incomplete resection, AEs, and advanced histology on final specimen necessitating careful selection of endoscopic resection technique. 19 In our study, submucosal fibrosis and HGD on final histopathology were associated with the inability to achieve technical success. HGD is associated with increased density of cellular structures, loss of normal glandular pattern, and progressive cellular changes with myofibroblast proliferation in the lamina propria. 20 21 Hypothetically, these changes may impede mucosal layer floatation underwater and lead to technical challenges during resection. En-bloc resection of giant colorectal polyps is challenging with h-EMR. 22 In a recent prospective randomized controlled trial, comparing uEMR with h-EMR for LNPCPs between 20 and 40 mm in size, en-bloc resection for polyps >30 mm in size was 0% in h-EMR cohort compared to 15% in uEMR cohort. 13 Previous studies have demonstrated improved R0 resection, lower incomplete resection rates, recurrence rates and procedure times for uEMR compared to h-EMR for polyps up to 40 mm in size. 8 13 Currently, there is limited information regarding the efficacy of uEMR for removal of giant colorectal polyps.

Conventional EMR of large polyps can be associated with higher recurrence with rates up to 22% at six months, with polyp size >40 mm being a significant predictor of recurrence (OR:4.37). 23 24 Polyp size > 40 mm is associated with 3.5 times higher odds of recurrence after h-EMR. 25 Previously, a randomized trial demonstrated significant reduction in recurrence rate with uEMR compared to h-EMR (42.9% vs 6.3%, *p* = 0.03) for polyps 30-40 mm in size. 13 Our single-arm study of uEMR of 137 polyps with mean size 47.6 mm was associated with 8.7% recurrence rate at a median six months follow-up. 17 Nagl et al. had previously demonstrated a trend toward decreased recurrence rate (6.3%, 1/16) for polyps >30 mm in size when compared to polyps ≤30 mm (18.9%, 7/37), after uEMR suggesting added benefits in larger polyps. 13 The uEMR technique was associated with completion of resection with fewer pieces compared to h-EMR, suggesting that technique may allow leaving less residual polyp islands, leading to lower recurrence rate. 13 This could be one of the reasons for the low recurrence rate in our study. The initial benefit of uEMR was thought to be the high rate of en-bloc resection. However, our study and other reports suggest that it may actually be beneficial in piecemeal resection as well. The ability to grasp more tissue with uEMR likely leads to removal of larger polyps in fewer pieces and hence reducing islands of residual polyp tissue which in turn reduces recurrence. Similarly, the better tissue grasp with uEMR likely prevents snare slippage in flat polyps, which could also have been a factor for lower recurrence. In addition, saline immersion also allows for better visualization, which can lead to improvement in detecting small islands of residual polyp during index resection.

In comparison to uEMR, endoscopic submucosal dissection (ESD) is associated with a lower rate of recurrence. 25 En-bloc resection of giant colorectal polyps can be performed with ESD; however, ESD is time consuming, resource intensive, associated with increased AEs, and limited to centers with expertise. 26 Due to these limitations, universal ESD approach for all LNPCPs is not feasible in the United States, and uEMR is a valuable technique in the endoscopists’ armamentarium for removal of giant colorectal polyps.

Our finding that uEMR is safe for giant colorectal polyps is not surprising. Recently, prospective randomized trials of uEMR of LNPCPs 20–40 mm in size demonstrated a similar AE rate compared to h-EMR. 13 AE rates increase with the size of the lesion. Previous studies have reported an AE rate of up to 10.9% with h- EMR of LNPCPs ≥40 mm. 27 Larger polyp size, right-sided location, and anticoagulant use have been associated with increased risk of delayed bleeding. 28 29 30 Delayed bleeding was observed in 9.5% of cases in our study, and underlying fibrosis was associated with increased risk of delayed bleeding. Lower rates of prophylactic defect closure (66%) in our study, possibly due to large post polypectomy defects could contribute to delayed bleeding. Majority (77%) of delayed bleeding occurred in the right side (cecum up to hepatic flexure), and only 30% of them were prophylactically closed. In a landmark randomized controlled trial, Pohl et al. demonstrated that clip closure after EMR of large colon polyps prevents delayed AEs, especially in proximal polyps. 31 However, despite intention to treat, nearly 32% patients in the clip group (median polyp size: 30 mm) did not undergo complete defect closure in the study, highlighting challenges with defect closure for larger resections. Intraprocedural AEs can be managed endoscopically; however, delayed AEs can lead to hospitalization and repeat endoscopic or surgical procedures. Prophylactic closure of EMR defects can prevent delayed bleeding especially for right colon and large defects. 31 32

Our study is not without its limitations. First, the retrospective nature of the study introduces the possibility of selection bias in the initial choice of lesions for uEMR and limits the ability to ascertain complete follow-up data in all cases. The lesion selection criteria, technique, electrocautery settings, decision to close the defect, and defect closure technique were up to the endoscopist performing the procedure. The polyp size was based on visual estimate of the endoscopist performing the procedure, which may introduce the risk of misclassification bias in our study. Our study demonstrated no benefit of prophylactic defect closure after uEMR. Being an exploratory analysis, we did not perform an estimation of sample size required to detect a difference, and nonsignificant outcomes in the right colon is likely due to small sample size. Management of residual or recurrent lesion was up to the endoscopist performing the procedure and was likely influenced by local expertise and institutional protocols. There was a trend toward increased recurrence in patients with prior resection attempts, submucosal fibrosis, and decreased recurrence with margin ablation; however, an association could not be proven, likely related to the study being underpowered to detect a difference. Recurrence rates were assessed at a median of six months post procedure, and long-term follow-up data after uEMR is required to confirm efficacy of the technique. Furthermore, being a single-arm study, direct comparisons to alternative techniques such as h-EMR and ESD could not be performed. In the study, definition of delayed AEs required intervention or hospitalization; hence, AGREE grade I events are likely not captured. Patients with mild hematochezia or post-electrocautery coagulation syndrome who underwent conservative management as outpatient are likely to be under-represented. Due to the low number of events (recurrence and delayed bleeding), multivariable models were limited to a small number of covariates in accordance with accepted events-per-variable principles, which limited the ability to estimate predictors of recurrence and delayed AEs. Despite these limitations, this is the largest multicenter study to our knowledge evaluating outcomes of uEMR for removal of LNPCPs ≥40 mm in size. Our analysis included a wide range of morphology and histology, along with the variations in procedural technique and operator experience, which reflects real-world experience and make the study outcomes generalizable and adoptable in community practice.

In conclusion, our study demonstrates that uEMR is technically feasible for the removal of giant colorectal polyps having an acceptable safety profile and recurrence rate. Future prospective studies comparing uEMR to h-EMR should be performed to assess outcomes to evaluate the superiority of the technique for managing giant colorectal polyps.

**Abbreviations**
uEMR: Underwater endoscopic mucosal resection
LNPCP: Large non-pedunculated colorectal polyp
SAE: Severe adverse event
HGD: High-grade dysplasia
CRC: Colorectal cancer
USMSTF: United States Multi-Society Task force
EMR: Endoscopic mucosal resection
h-EMR: Hot endoscopic mucosal resection
TTS: Through-the-scope
DAT: Dual Action Tissue
OTSS: Over-the-scope suturing system
TTSS: Through-the-scope suturing system
ASGE: American Society for Gastrointestinal Endoscopy
AGREE: American Society for Gastrointestinal Endoscopy adverse events in GI endoscopy
ESD: Endoscopic submucosal dissection
